# Attitudes of nursing faculty members toward technology and *e-learning* in Lebanon

**DOI:** 10.1186/s12912-021-00638-8

**Published:** 2021-06-30

**Authors:** Rona Nsouli, Dimitrios Vlachopoulos

**Affiliations:** 1 EdD Online Programme, School of Histories, Languages and Cultures, University of Liverpool, Liverpool, UK; 2 Digital Society School, Faculty of Digital Media & Creative Industries, Amsterdam University of Applied Sciences, Amsterdam, Netherlands

**Keywords:** Information Communication Technology (ICT), Attitudes, Nursing, Teaching, Learning, Mixed Methodology Research (MMR)

## Abstract

**Background:**

Our transition to an “information society” means that Information and Communication Technology (ICT) has become integral to our lives. ICT has also become an essential aspect of medical institutions and healthcare settings. Healthcare professionals, especially nurses are required to use ICT in their daily work. In Lebanon, however, due to political factors, many universities have not introduced technology or any form of ICT in their curricula. Institutions of higher education do use technology in various ways, however, successful incorporation of ICT in education requires acceptance by instructors who are expected to use ICT in teaching practices. Although international findings reveal that ICT should be used in nursing education, some faculty members experience difficulty integrating it.

**Method:**

A mixed methodological research approach was used to investigate the attitudes of nursing teaching staff toward the use of ICT in nursing education.

**Results:**

Our findings revealed three categories of faculty with differing attitudes to the use of ICT in teaching and learning: *pioneers*, faculty members who have developed positive attitudes toward ICT usage; *followers*, faculty members with neutral attitudes; and *resisters*, faculty members with negative attitudes.

**Conclusions:**

Identification of the nursing faculty members’ attitude toward ICT and the challenges faced by them contributes to the integration of ICT into nursing curricula and further development of educational practices.

## Background

The development of a nation depends on the education system it provides to its citizens; those citizens are the future leaders. As instructors continue to be facilitators of teaching and learning, students enrolled in different educational institutions reflect the society. Alemu [1] mentions that it is essential for instructors to be prepared and equipped with skills to meet the requirements of the current generation, especially in the use of information, communication, and technologies (ICTs), and it should be integrated into their teaching practices.

ICTs are essential forms of literacy that contribute to higher education in the 21st century [2]. Researching and communicating in a digital environment has become an important form of academic literacy [2] and ICT is being used in higher education institutions in the hope that the technology will “promote inclusion, stimulate innovation and improve efficiency” [3] (p.557). However, a significant challenge in using ICT in higher education is to find the means to encourage faculty members to use it.

Lebanon’s education system is dominated by private institutions. The teaching methods between organizations vary significantly, and there is little consistency among different programs and disciplines [4]. Over the past years, Lebanon has suffered severe political instabilities, preventing the upgrade of its education systems on a national level. The government has always had competing priorities, with the recent one being the two million refugees fleeing from the Syrian war and seeking shelter in extremely poor environmental conditions [5].

Further, nursing is not a domain that attracts many students; as a result, the nursing department receives low funds and minimal research is being conducted on nursing curricula and teaching practices. While nursing faculty members are expected to keep pace with the changes happening in both settings—healthcare and higher education— the reality is more complex.

The purpose of this study is to investigate the digital profile and attitudes of nursing of nursing faculty members on the use of ICT in teaching and learning. Specifically, it will assess their familiarity with the use of ICT and it will reflect on the technologies needed for a 21st century nurse. Finally, the study will analyze the challenges faced by nursing faculty members when it comes to use ICT in nursing education and will explore the need for training and professional development opportunities on quality nursing education through the use of ICT.

### Literature review

This section provides a conceptual investigation of the concept of “attitude”, which justifies the use of the term throughout this study. Moreover, the impact of ICT on students’ learning and achievement will be presented with a focus on e-learning and the major ICT developments in the health sector and nursing education. The section ends with a reflection on the current situation in Lebanon as far as the use of ICT in nursing education is concerned.

#### Importance of attitudes

Davis [6] argues that attitudes are feelings of favorableness or unfavorableness toward a system; the usage of technology is individual by nature, and system usage is not driven by social influences. Previous assessments of faculty members indicate that little is being done to support faculty during their transition toward using more advanced methods in the workplace [7]. It is important to study faculty attitudes toward the use of ICT because these—positive or negative—are considered beneficial for individuals [8–10].

Goodwin et al. [11] emphasize that the personality traits of teachers may affect how they value ICT. Being an ICT competent individual reflects a form of technology acceptance, which is translated into the use of ICT in classrooms. Another factor to be considered is the “importance” that teachers place on the use of ICT. The level of importance is reflected in cultural perceptions [12]. If the culture requires or encourages the use of ICT, the teachers are more willing to take the required risks.

When faculty members consider themselves valuable to their institution, or the institution provides them with enough incentives to work with a positive impact, they are more willing to invest in themselves and update their practices accordingly [11, 13].

Thus, according to Costello et al. [14], institutions of higher education play a significant role in the preparation of health science graduates entering the workforce. Chan et al. [15] found that healthcare consumers expect the staff to have knowledge-based training, technological expertise, and clinical competence.

#### Impact of ICT on students

Students’ engagement with ICT in educational settings could comprise the learning content management systems (LCMS) of the institutions, e-journals, and search engines, plagiarism detection sites like Turn-it-in; and individual daily use of the Internet, social media, and emails [16–18]. Students use a variety of digital devices, particularly smartphones and PCs. According to Henderson et al. [16], students’ engagement with digital technology can be viewed from two perspectives—logistics and learning.

Prenski [19] describes current students as *digital natives*, for they have been born into a digital world and take technology for granted. They spend significant amount of time communicating, learning, and playing games using digital devices [20]. *Digital natives* are described as being technology savvy; for the use of technology seems easy for them. They are capable of keeping pace with the fast rate of technology advancement, are enthusiastic to learn, and are able to integrate technology easily into their daily lives.

#### Relevance of e-learning

The use of ICT also facilitates learning both on- and off-campus. While on-campus learning means that a student is physically present in a classroom during teaching time; off-campus entails teaching and learning activities that do not require students to attend campus, often referred to as *e-learning*” [21]. Usually, *e-learning* supplements rather than replaces on-campus education [22]. Depending on the context, the terms *hybrid* or *blended* learning are used, whereby instructors can introduce technology gradually. This might be the first step in settings in which traditional teaching approaches persist. Instructors would start using ICT in their teaching practices while building effective learning experiences [23].

Educators need to maintain a balance between the design and implementation of the resources involved in *e-learning*. Although Voutilainen et al. [24] mention that the goal behind the development of *e-learning* is to develop online studies that are more student-oriented, the success of *e-learning* methods is highly situational. *E-learning* has become an integral part of health professional education, and its multiple benefits have been identified, including increased access to education, cost-effectiveness, and accommodation of multiple learning styles. Despite the need for a shift toward the use of ICT, there are still very few cases of *e-learning* in undergraduate nursing programs [22]. Additionally, there are still significant variations in *e-learning* quality [25].

#### Impact of ICT on the health sector

ICT has become an essential tool in the health sector [26]. Although there are multiple differences in terms of how institutions within the same country and across continents apply ICT in healthcare settings, the healthcare sector is undeniably shifting to a more technology-oriented setting [26]. According to Meier et al., [27], *e-health*—the use of ICT in health—is expected to improve the efficacy and efficiency of healthcare. The healthcare sector is one of the largest service-oriented sectors in the world, hosting numerous clients and run by an army of health professionals [28]. One of the largest of these is the nursing body. Current research [29] verifies that telenursing is growing, in view of its presence in different countries, with strong evidence and benefits of its use. Avison and Young [30] found that hospitals, polyclinics, and private centers are shifting from paper-based to e-health or patient electronic systems. While there is limited evidence available about the effectiveness of such solutions [31], preliminary research results demonstrate that such implementations improve the quality, efficiency, and accountability of health institutions [14].

According to Skiba et al. [32], nursing schools will soon experience a digital divide. Faculty who are not aware of or who do not have access to clinical information systems—e-health systems in healthcare settings—will have difficulties in understanding the changes needed in the nursing curriculum while the other staff will keep pace with ICT use. According to Button et al. [33], nurse educators are expected to integrate digital technologies to facilitate the learning process of their students. With the lack of appropriate preparation of nursing students in clinical settings, there is a chance of a mismatch between the available technology in educational institutions and healthcare institutions [34]. The use of technology in today’s health education programs is an essential learning opportunity for students, which will first, enhance the preparation of future clinicians, and second, facilitate the development of professional skills that will prepare them for future workplace settings [34]. Costello et al. [14] mention that technology use in health education should include—patient simulation, support for inter-professional team functions (realizing the role of each member of the healthcare team), training in the use of health informatics, using social media to enhance communication, and improving communication literacy. Students, educators, patients, and stakeholders are required to gain new skills more than ever before, so that they are up to date and effective [34]. Pilcher and Bradley [35] emphasize that educators are required to increase their understanding of technology use. Further, effective educators should grasp the attention of today’s students and develop methods to keep them engaged in their education, including the use of technology [10, 36, 37]. Either face-to-face or through online learning, educators should involve students in active learning that fosters interaction and connectivity [35].

#### Relevance of ICT to nurses’ competencies and skills

Previous attempts to implement digital health interventions have demonstrated that an informed, engaged and skilled clinical workforce is essential. There is therefore a necessity to support clinicians in gaining experience in digital health approaches, and nurture the career pathways of those who show an early interest [38]. Specifically nurses are frequently monitored for their skills and competencies, often involving the use of advanced machinery related to patient care. The simplest of these is the electronic medical records or individual patient charts in the form of i-pads. Nurses are required to have the necessary skills to manage different ICTs in healthcare settings.

Nurses complain of a gap in their informatics knowledge and skills [39]. Relevant research [40] also confirms that only few nursing programs prepare students to use this technology in providing direct patient care. Nursing faculty members are required to teach students these competencies and prepare them for ICT-related practices in healthcare settings. However, up to 75 % of nurses entering the workforce report learning their ICT skills during on-the-job training in healthcare settings and not in training institutions [41].

#### Context of Lebanon

Nurse educators in Lebanon are updating the educational curricula to integrate ICT. They need to identify institutional and other resources that are essential for them to develop positive attitudes, enhance existing skills, and build new ICT competencies. There is evidence of innovation in teaching methods. Kantar and Massouh [42] document the successful use of case studies in nursing education in Lebanon. Hoffart et al. [43] document the use of active learning methods through storyboarding to minimize the gap between the theory and clinical experiences of nursing students. Fawaz and Hamdan-Mansour [44] discuss simulation to enhance the clinical experience of students, with nursing educators reporting flexibility in designing and evaluating learning experiences.

The Lebanese healthcare system is described as an aging system because 65 % of patients seeking healthcare services in the country are geriatric. Thus, there is a need to facilitate healthcare services for this population using ICT. Lebanon has the required capacity, innovation, and skills, however, the lack of adequate infrastructure and regulations has slowed the development of ICT in hospitals [45]. Personnel working in clinical settings have identified challenges in shifting toward the use of ICT, particularly the lack of knowledge. Resistance to the adoption of ICT by senior staff in healthcare settings is well-documented and has negatively impacted the attitudes toward the use of ICT [45].

The above-mentioned studies acknowledge innovation in teaching practices in nursing education. However, there is limited research on the use of ICT in this field and the attitudes of faculty members toward technology, despite the multiple calls for curricular transformations due to the inadequate preparation of graduates [46].

As a profession, nursing, in Lebanon, has the potential to attract students who seek majors that will challenge their critical thinking and offer stimulating educational experiences. Although healthcare domains are specialized, having updated curricula and innovative teaching approaches, would build a better generation of students on multiple levels. To do this, we need to look at our attitudes toward change in general, and toward technology and ICT more specifically. As a profession, nursing is not regarded as an honorable occupation, and does not attract numerous applicants [47]. Additionally, nursing schools and departments within higher educational institutions do not always receive the required funds, and this affects multiple areas involved, such as libraries, computer labs, and even clinical settings.

## Methods

This research applied a mixed methodology approach (MMR). Specifically, the design was composed of two distinct phases of quantitative and qualitative data collection [48], following a convergent design. Although the convergent design adopts a concurrent data collection approach, data collection was sequential with quantitative preceding qualitative [49] to minimize the interaction between the two data sets.

The main purpose of the combination of data collection instruments was to demonstrate that triangulation by distinctly different methods—questionnaires, and interviews—can lead to confirmation of findings and explain the circumstances that allow this to occur [50, 51]. The data collection instruments were used to complement each other.

Voluntary sampling was used for the selection of participants [48], as they willingly volunteered to participate in this research. Four or five faculty member volunteers were required from each institution. The same participants who volunteered to complete the survey also took part in the interview. The participants were selected on a first-come-first-served basis so that no one was intentionally eliminated.

Nursing schools in Lebanon do not consist of large departments with numerous faculty members. The programs often rely on part-timers and adjunct faculty for teaching. As a result, having four to five participants from each department constituted a relatively suitable number. Eighteen participants took part in this project, sixteen females and two males, and the participants constituted an adequate representation of their institutions, which enhanced the possibility of generalizing the findings of the data [51].

The quantitative data collection took place once the volunteer participants were identified. Four private institutions of higher education that offer a three-year Bachelor of Science in Nursing (BSN) program, and use English as the medium of instruction, took part in this study for convenience and standardization. The researcher delivered sealed envelopes (containing the survey, consent form, and participation information sheet) to their offices. The researcher fetched the completed surveys after approximately one week. The researcher received approval from the Institutional Review Board for each institution that participated. Once that was achieved, the Virtual Programme Research Ethics Committee (VPREC) at the University of Liverpool approved commencement of the project.

The qualitative data collection method involved a one to one interview with the participants in a convenient setting at their institutions. The researcher started the interview by greeting the participants, obtaining approval for the use of a recorder, and then proceeding with general questions and moved toward the specifics, focusing on the attitudes of faculty members toward the use of ICT. The interview duration was approximately 45 min, and the meeting was in a private room with closed doors. Toward the end of the interview, the researcher thanked the participants for their time and answered any inquiries.

Quantitative data followed an analysis using descriptive statistics. Descriptive statistics designate the findings so that researchers can interpret and analyze what they represent [52]. Qualitative data is analyzed using a thematic analysis approach, which remains the most common method of data analysis [53]. Thematic analysis refers to the method of identifying themes from the qualitative data. Table 1 below, illustrates the thematic analysis process with the identification of themes and subthemes, as they emerged from the interview questions with some supporting quotes as examples.

**Table 1:** Themes, subthemes and supporting quotes illustrating the thematic analysis process, following the example of Burnard [54], Gallagher and Porock [55], Priest, Roberts and Woods [56], and Woods, Priest and Roberts [57]

Themes and Subthemes Table			
Data collection Interview Questions	Themes Identified	Subthemes Identified	Supporting Quotes
How is ICT influencing education and the new generation of students in general? What is the purpose behind the use of ICT? What do you think of ICT in nursing education? How has technology influenced the nursing profession?	Perception of Interviewees on ICT	Enhances the Learning Experience of Students	“Some students are passive learners, they rely on what they are given, read their book and PPP and that’s it for them. The use of ICT is helping those students become active learners and self-directed”.
		Enhances Access to Information	“Knowledge is advancing quickly, everyday there are new guidelines, if we want students to apply the most recent practice, we should be able to access the most recent update”.
		Decreases the Gap Between Students and Teachers	“Teachers have become closer to students using ICT, because the use of ICT attracts students further to their studies”.
		Challenges Faced When Using ICT	“Part of the challenges in this country include slow internet, and electricity shut downs, these alone do not encourage the use of ICT”.
Do you think nursing students need to be prepared to use ICT? Why? How? Should the nursing curriculum include computer training for students? If Yes/ No, why? In your opinion, for what purpose nursing students use ICT? Do you think current nursing practice requires the use of ICT in health care? How do you foresee the role of technology in nursing education in the future?	ICT Use	ICT as a Communication Method	“Due to the compromised security in Lebanon, sometimes we can get to communicate with students rapidly and directly through text messages and WhatsApp”.
		Contexts in Which ICT Is Used	“Students get their personal computers (PCS) to class to record lectures and take their notes electronically”.
		Consistency in ICT Use	“We don’t use ICT in a standardized manner, we need to standardize its use so there would be better consistency. We have the university electronic system, we don’t all use it in the same way, while some use a lot of its features, still others are bound to the basics”.
		Usage of New Technology Approaches	“I will use a new approach, if it is accessible, I already downloaded lots of videos for my classes like on suturing and some other specific procedures”.
		ICT in Simulation Labs and Electronic Libraries	“Using electronic libraries also encourage students to access and apply evidence-based practice, we need to reach information to know what is happening”.
		Discrepancies in ICT Use	“Using ICT regularly requires extra personal efforts. While I am enthusiastic to do it, this is something that not all faculty are passionate about, students decide how and what they want to learn, and we try to meet their needs”
Are you willing to try new teaching approaches in your classroom related to using ICT? How? Do you think you are using enough ICT with your students? In your opinion what are the factors that encourage the use of ICT? What are the factors that discourage you from using ICT?	ICT Knowledge	Need for Further Training	“There should be updates and workshops for faculty members, as we feel that students know more”
		Presence of ICT Policy	“Technology has a major role in our practice and we use it a lot; however, there is no clear policy on ICT use due to varied reasons”
		ICT Influence on the Nursing Profession	“The future of the nursing profession, and nursing as a career is changing, in which ICT is becoming a major part of it”
		Competencies and Skills	“Nursing students need to have ICT skills, because hospitals recruit nurses from different backgrounds, and there are varied ICT skills levels among students, they all should be well prepared to practice”

 For ethical considerations, this research project was reviewed by the VPREC at the University of Liverpool. Further, three concepts of ethics have been considered and maintained in this project: anonymous, confidential, and autonomous [58].

## Results

This research employed mixed methodology, and qualitative data was dominant. The quantitative analysis followed a descriptive analysis, while the qualitative data followed a thematic analysis. The results of both datasets are discussed in the following sections.

### Quantitative results

The questionnaire was composed of six sections, with the first section addressing demographics. Each section had a distinct set of questions revolving around different goals. Demographically, the sample consisted of eighteen participants, sixteen females and two males, who worked at four different nursing departments in four institutions of higher education. The dominant age range was 25–44 years (Table 2).

**Table 2:** Sample’s socio-demographic characteristics (*n* = 18)^a^

	Number	Percentage (%)
**Age (years)**		
25–34	8	44.4
35–44	8	44.4
45–54	1	5.6
55–64	1	5.6
25–34	8	44.4
35–44	8	44.4
45–64	2	11.1
**Gender**		
Female	16	88.9
Male	2	11.1
**Education level**		
BS	2	11.1
Master	12	66.7
PhD	4	22.2
**Position**		
Assistant Professor	4	22.2
Lecturer	5	27.8
Instructor	8	44.4
Other-part-timer	1	5.6

Note: ^a^Numbers do not add up to 18 because multiple answers could have been selected

The findings revealed that exposure of most faculty members to ICT and their ICT knowledge are based on their individual interest in ICT. Those who reported feeling competent in ICT attribute it to their personal interest in ICT (Table 3). All participants reported using different ICTs, in addition to different hardware and software devices. Most participants used email and the Internet. Most participants also reported using the LCMS, available at their institutions, although not all of them reported using the same platforms equally (Table 4).

**Table 3:** Familiarity with the use of information, communication, and technology (*n* = 18)

	Number	Percentage (%)
**ICT knowledge**		
Seminar attendance	4	22.2
Training programs at university	3	16.7
Practice	10	55.6
No ICT knowledge	1	5.6
**Frequency of computer use at home**		
Once per week	5	27.8
Once per day	1	5.6
Various times per day	12	66.6
**Frequency of computer use a work**		
Various times per day	18	100.0
**E-mail address**		
Yes	18	100.0
**Perceived familiarity with ICT**		
Expert user	2	11.1
Good user	10	55.6
Average user	5	27.8
Weak user	1	5.6
**Reasons for considering being expert/good ICT user**		
Training courses attended with organization	3	0.25
Personal interest	9	0.75
Specialization/academic background	3	0.25
**Reasons for considering being average/weak ICT user**		
No training	5	0.8
Not compulsory at work	1	0.2
No interest	0	0.0

**Table 4:** Types of ICT used in nursing education (*n* = 18)^a^

	Number	Percentage (%)
**ICT use with students**^**a**^		
Word processor	3	16.7
Presentation software	14	77.8
Using the internet	5	27.8
Using other applications	1	5.6
**Student use of ICT in lessons**^**b**^		
Using subject specific software	5	29.4
Studying for exams	10	58.8
Using internet to search for information	14	82.3
Collaborating between classrooms	4	23.5
Working on projects	15	88.2
Solving problems, making decisions, or forming opinions	5	29.4
Students don’t use ICT in class	1	5.8
**ICT use at work**^**a**^		
Personal use	11	61.1
Work related administration	12	66.7
Recording marks on a spreadsheet	14	77.8
Typing exam papers	16	88.9
Finding information and resources on the internet	17	94.4
Accessing resources using online databases	15	83.3
Developing teaching material	11	61.1
Developing content for student use	15	83.3
**ICT use at home**^**a**^		
Personal use	15	83.3
Work related administration	7	38.9
Recording marks on a spreadsheet	7	38.9
Typing exam papers	10	55.6
Finding information and resources on the internet	16	88.9
Accessing resources using online databases	11	61.1
Developing teaching material	8	44.4
Developing content for student use	8	44.4
**Participants with the ability to**^**a**^		
Use a memory stick to transfer data	14	77.8
Install new software on a computer	15	83.3
Install a printer	14	77.8
Solve basic technical problems	7	38.9
Log in to a network	15	83.3
Add a shared folder on a network	12	66.7
Make information on a network secure	6	33.3
**Technologies used for teaching purposes**^**a**^		
Video	6	33.3
Digital cameras	4	22.2
Data projectors	14	77.8

Note: ^a^Numbers do not add up to 18 because multiple answers could have been selected

^b^Percentages obtained by dividing over 17 because of one missing answer

The faculty members further highlighted the role of institutions in enhancing the choice of technology by creating a motivating climate, advancing the necessary ICT policies, and developing the needed infrastructure in classrooms. The faculty members considered the need for further training in ICT-use to be essential and recommended such training to be made compulsory. All faculty members considered ICT to be fundamental for their work and essential for nursing education; hence, the majority do teach their students about copyrights, plagiarism, and referencing (Table 5).

**Table 5:** Training needs on the use of ICT in nursing (*n* = 18)^a^

	Number	Percentage (%)
**Perceived need to include ICT training in nursing education**		
Yes	17	94.4
No	1	5.6
**Received ICT training covering**^**a**^		
Workplace administration	2	11.1
Computer literacy	6	33.3
Using subject specific software	7	38.9
Teaching ICT as a subject	2	11.1
Finding and using resources from the internet	7	38.9
Planning lessons or projects that integrate ICT	4	22.2
Providing technical IT support at work	4	22.2
**Teaching students about**^**a**^		
Copyright	10	55.6
Plagiarism	11	61.1
Referencing	10	55.6
**Consider training nurses on ICT to be fundamental for teaching practice**		
Yes	18	100
**Training seminars should be**		
Compulsory	13	72.2
Optional	5	27.8
**Most effective training method**		
Short face-to-face courses	4	22.2
Face-to-face courses provided by the university	6	33.3
Mixed system (part online, part face-to-face)	8	44.4
**Training session preferred timing**		
During working hours at the university	15	83.3
Outside working hours	3	16.7
**Main focus of training**		
Theoretical knowledge about ICT use in education	2	11.1
Practical tips and hands-on activities on ICT use	4	22.2
Combination of both	12	66.7

Note: ^a^Numbers do not add up to 18 because multiple answers could have been selected

Regarding *e-learning*, the participants reported an absence of clear guidelines and that *e-learning* is minimally used. Online learning is not acknowledged by the Lebanese Ministry of Education and Higher Education (MEHE), hence the resources required for upgrading educational methods to use *e-learning* [59] are not available. However, some of the faculty reported the use of traditional teaching methods such as blended learning, problem-based learning, and flipped classrooms in addition to simulation labs (Table 6).

**Table 6:** E-learning (*n* = 17)^a^

	Number	Percentage (%)
**Institutional strategy on the use of e-learning**		
No	10	55.6
Underdevelopment	2	11.1
Some faculties have their own strategies	2	11.1
Yes	3	16.7
**Institution use of e-learning**^**a**^		
No	7	38.89
Only recently introduced	4	22.22
Some individual teachers use it	2	11.11
Yes, some departments use it	2	11.11
**E-learning should be used in nursing education**		
No	1	5.56
Maybe	3	16.67
Yes	14	77.78

Note: ^a^*n* = 15 because of 3 answering “Don’t know”

### Qualitative results

The thematic analysis of the qualitative data followed three main themes about ICT: Perceptions of participants, use, and knowledge.

#### Perceptions of participants toward ICT use

Most of the participants perceive that ICT is important for nursing education and is being increasingly integrated into contemporary nursing curricula. Some of the participants considered that the importance of ICT lies in the fact that it has shifted nursing practice in clinical settings and has contributed to better outcomes in patient care:

“ICTs have shortened patients’ hospital stays” [Randa].

ICT is also important for students’ learning. This generation of students is interested in technology use and find it helpful in their studies. ICT improves individual learning and encourages students to work on their own for further exploring their educational needs, thus enhancing their self-learning abilities.

ICT use has created easy access to information, especially with the availability of the Internet and Wi-Fi services. There are simulation labs available for students to use. E-books are replacing hard copy books, students submit assignments through university portals and emails, and in most nursing environments, evidence-based practice dominates with new recommendations and research. Students use ICT to build their knowledge and to check for new evidence-based practices, and recommendations. ICT helps students develop their computer skills:

“Now students study on their PCs and submit assignments through emails. They learn through technology” [Lama].

Faculty members also reported that exposure to new teaching approaches using ICT gives them self-satisfaction, especially when they feel that students are more engaged. Some of them have attended training workshops to use new and advanced teaching methods. This is evident in the following statement:

“I recently attended a workshop on the use of social media and educational technology to update my teaching practices” [Randa].

Further, ICT enhances access to global and international information and bridges the gap between students and teachers:

“Teachers have become closer to students using ICT because the use of ICT attracts students further to their studies” [Samer].

From another perspective, faculty members raised many challenges in using ICT, which prevent it from being used optimally. Lebanon, as a country, suffers from programmed electrical shutdowns. The Internet is one of the slowest in the world, and limited Wi-Fi services are available for faculty and students.

Infrastructure challenges are experienced in the country and by universities; for example, Internet access may be unavailable to institutions or classes. There are problems with the maintenance of hardware devices such as computers, printers, and LCD projectors. Another limitation is that not all students have laptops, home Internet services, or back-up generators at home. Further, ICTs are costly and require set budgets.

An important finding provided by this study regarding faculty attitudes is resistance. Some staff members are afraid that their roles will be eliminated with the use of ICT. They believe that nursing programs are already condensed and that the use of ICT will add to their workloads and create further time constraints. Some faculty members also mentioned that students are not always capable of using ICT safely, because they can access the wrong resources and information.

“We are overloaded with other things, we end up not using ICT, in addition we need workshops to master it” [Rawan].

“It is a bit scary, when students google wrong information, in our domain there is no room for mistakes, small things can be fatal” [Sara].

#### ICT use in nursing education

There are multiple purposes behind the use of ICT in nursing education. Whether faculty members like it or not, and whether it is available for them or not, ICT use has become a part of the educational system and a lifestyle for some. In nursing education, ICT use has created better facilities for teaching and learning. More precisely, it has become easier to transfer theoretical knowledge into more practical and hands-on information.

“Students can see 3D images of body organs now, a thing that was not available for the previous generation” [Salem].

“Use of evidence-based research is the essence of the use of ICT for me” [Lea].

ICT is an essential communication method/tool between teachers and students, between faculty members themselves, and between health professionals. Different social media applications such as WhatsApp and Facebook are used to enhance communication with students:

“Due to the compromised security in Lebanon, sometimes we can communicate with students rapidly and directly through text messages and WhatsApp” [Salwa].

Smartphones are frequently used in contemporary nursing education. More than one participant reported that they encouraged students to download nursing applications, especially those related to medication calculations and electrocardiogram readings.

In nursing education, ICTs are used in classroom and clinical settings. ICTs are widely used in classrooms and classroom-related activities in a variety of teaching methods. They can be in the form of hardware installed in classrooms such as computers, LCD projectors, videos, and cameras. The software includes programs such as MS Word. Services or platforms include Wi-Fi and the Internet. When it comes to clinical settings, the ICTs include health information systems (HIS) and devices that nurses use while working with patients. Faculty members consider that ICTs have changed the approach to education, and believe that students are interested in using ICT.

“ICTs have changed the way of education … students are obliging faculty members to use ICT in their courses … students ask for more technology” [Reem].

ICTs have changed the way students study. While the traditional image was of students holding and reading books, the new images include students holding laptops and smartphones. University electronic systems or portals help students prepare for classes through uploaded lectures, provide alternative means of communication with their instructors when required, and enable them to submit assignments online. University portals serve students’ agendas:

“All information related to students is available on the university portal …” [Reem].

In settings where university electronic systems are not available, social media plays a replacement role in which faculty members develop closed Facebook pages or WhatsApp groups to communicate with students. ICT use can create better teaching consistency among instructors when they all use the same platforms. It can also encourage faculty members to use different teaching approaches, which are enhanced by the choice of ICT. The dynamics of the classroom depend on students and how they respond to teaching approaches.

“Students are more active in the classrooms with the use of ICT and when they go find information” [Salwa].

The most important factor that affects teachers’ choice of teaching approaches is students’ interest and responses to the new method.

“Each time I try something new I ask students to evaluate, and their comments come like we should do this more often. Students are never satisfied, they always want more of technology use. For me, I aim at trying something new each year” [Reem].

To summarize, ICT is used for multiple purposes, the most common of which is communication. ICT has eased communication on multiple levels among faculty members and students, and among nurses and different health professionals. Social media platforms are used to communicate with students and send them study-related information. ICT is used in simulation laboratories and electronic libraries, and these have contributed positively to students’ learning. ICT is used in classrooms and clinical settings, using different hardware and software but there are discrepancies and inconsistencies in its usage: while some faculty members feel that what they do is not enough, others use ICT out of personal interest.

#### ICT-related knowledge

The participants responded differently to the question of how they had gained their ICT knowledge. While some had attended training workshops, others stated that their ICT knowledge was based on self-interest and independent learning. However, there were two evident means of gaining ICT knowledge—self-directed learning and institutional requirement. Some participants considered that ICT use enhanced personal growth:

“In our context, it is a self-directed act … lots is based on personal efforts” [Reem].

Many faculty members reported the need for extra training and workshops on the use of ICT. They feel that sometimes students are “ahead of them” regarding technological applications. The participants expressed the need for someone to teach them to acquire “hands-on” experience when it comes to building ICT knowledge. There is no definite ICT policy in the institutions that were part of this study. However, in institutions where there is a an LCMS or a university portal for faculty and students, the faculty are required to use some of its features. The features include posting course materials, grades, course evaluations, and, in some cases, extra reading materials. Similarly, when it comes to online education, although most institutions offer online courses, there are no online degrees. The Ministry of Education/Higher Education (MEHE) still does not recognize online degrees, and as a result, most institutions have not developed specific policies regarding it.

ICT has recently influenced the nursing profession in multiple dimensions. Changes are occurring in healthcare settings with the transition from paper-based systems to electronic health records. New machinery is being introduced, and there are changes in the type of work that nurses perform. Communication patterns among health professionals are also changing with the usage of ICT.

*ICT competence* is not clearly defined in nursing terminology, as its aspects have not been specified. In this study, there was a consensus that nurses need to be able to use technology, computers, and the Internet. In general, nurses need to be able to manage the diverse types of technology available, research skills, and access new recommendations for practice. They should be able to function with and around technology because it dominates most contemporary healthcare settings. This puts pressure on faculty members who need to develop ICT skills to ensure that their students also acquire workplace-relevant ICT skills.

## Discussion

The literature has identified multiple factors that affect teachers’ attitudes toward the use of ICT. These include access to resources, the quality of software and hardware available [9]; attitudes toward technology, ease of use of technology [10]; educational background and beliefs, incentives and motivation, self-efficacy beliefs [11]; support from colleagues and university administration, school and national policies, commitment to continuous learning and updates in teaching methods [16]; and the training background of educators [36].

This study further elaborated that stress, lack of experience, lack of knowledge, limited skills, and poor infrastructure, are factors that prevent educators from using ICT in their teaching practices. The results of this study demonstrate that nursing faculty members’ attitudes can be categorized into three groups: faculty with positive attitudes who are *pioneers* in the use of ICT, staff with neutral attitudes—who tend to be *followers* by nature—and faculty members with negative attitudes—*resisters* who oppose ICT use [14].

The *pioneers* in the use of ICT are those faculty members who have developed positive attitudes toward its usage. They are *pioneers* and embrace novel approaches in education that emphasize the use of ICT. Critically, they acknowledge the importance of ICT in nursing education and healthcare practice currently and in the future. This is evident in the report that they have gained ICT knowledge by “doing their own” training. In other words, their competence is based on personal efforts and interest in using ICT. Institutions play a role in enhancing the choice to use ICT, when they create time flexibility and provide training workshops, but the faculty considers that their personal interests encourage them to use ICT. In scenarios where ICT knowledge was gained through personal interest, participants considered it to be a self-directed act, and that it developed from their passion to learn and use technology further in their teaching practices.

In some institutions, faculty members attended training workshops on teaching approaches such as the *flipped classroom method*, *team-based learning*, and *active learning*. Faculty members reported that each time they attended a training workshop, they tried to apply what they had learned, to see if it was applicable or not and to monitor students’ feedback. Some participants reported that they teach ICT-related courses and have ventured into knowing and applying ICT-related educational practices. Others rely on social media to transmit messages and share information with students. A participant reported that she developed groups on social media for each one of her classes to ease communication with her students and share materials and announcements. This level of commitment proves that these faculty members are *pioneers* in ICT use. They emphasize the importance of *e-learning* and admit that it creates flexibility for nurses who wish to excel in their careers while *e-learning* can provide time flexibility for nurses who work on a shift basis. Some instructors use self-guided applications, case studies, and wiki sites. *Pioneers* believe that success requires them to take risks and try new learning approaches. *They* also acknowledge that ICT constitutes an important step in the future of healthcare. They accept the reality that nurses must use ICT in different healthcare settings, especially with the advances happening on multiple levels such as robotics surgery, advances in healthcare machinery, and health information systems.

*Followers* are the second category, consisting of staff members who have neutral/passive attitudes. They admit that “ICT is the future”, but they do not make any extra effort to enhance ICT integration. *Followers* acknowledge the importance of ICT and use it only as required. For example, they use LCMS to meet the basic requirements of their institutions and keep pace with the technological requirements of their students. This group highlights the challenges they face and rely on institutional policies to advance ICT use. *Followers* do not feel that the ICT practices they know and use are sufficient to meet the interests of this generation of students. They believe that students are more knowledgeable than them in some areas, especially because this generation of students is interested and wishes to learn through ICT. *Followers* think that they need extra training to learn further about ICT practices and they rely on external support. They admit that communication and collaboration using ICT is faster and better, as is access to information and international research.

*Followers* use ICT at work for multiple purposes, the most common of which are: finding information, using the Internet, preparing exams, identifying resources, developing content for students, and recording marks. *Followers* also report using computer software at work, such as Excel, MS Word, and PowerPoint. They use the Internet regularly and send and receive emails. Most *followers* use hardware devices such as printers, photocopy machines, overhead projectors, scanners, and smartphones. However, these acts constitute the basics of the work that can be done with ICT. They understand updated research practices and acknowledge that ICT enhances access to research and recommendations. For them, the “Nursing Informatics” course, which is the only course on computer literacy provided to nursing students, is enough to prepare them for the workplace. *Followers* are interested in learning and trying new ICT-related teaching approaches, especially since they believe that traditional teaching approaches are no longer appealing to students. They admit that they are exposed to new teaching approaches though they need to be updated and trained. They feel that students are “ahead of them” when it comes to technology and ICT use, and they need to be able to keep pace with the demands of this generation.

*Resisters* are those faculty members who are against the use of ICT, other new forms of technology, or changes in the approach to teaching. *Resisters* are worried that ICT might eventually eliminate their roles, and they openly express a negative attitude toward its use. They further consider nursing curricula to be overloaded and believe that they do not have time to attend training workshops. If training workshops are provided, they wish to attend them during working hours and that they should be funded by institutions.

*Resisters* emphasize the challenges, such as the lack of consistency in ICT use, and tend to blame the institutions where they work for the lack of progress. They believe that institutions are required to provide a motivational climate and create incentives to encourage faculty members to use ICT. *Resisters* make “small steps” in the process of change. Without a clear ICT policy, *resisters* would not use ICT facilities or practices. Other constraints include a lack of funding, interest, and time. *Resisters* believe that ICT use requires extra effort to learn, and the curricula are already full. They do not like to use *e-learning* or social media, and reduce the role of social media to “applications to gather likes”. For them, the use of books and journals is essential for education and learning and cannot be replaced by e-books and e-journals. *Resisters* do not like the fact that students study using PCs and submit assignments via email. The faculty members do not use the required engines to monitor plagiarism. For them, ICT is a “time-waster”, because students can google the information they need, and get it “the easy way”, without referring to educational search engines and books. Some faculty members also reported resistance toward the use of new teaching methods. Another aspect that *resisters* highlight is the humane aspect of the profession. These faculty members feel that ICTs will eliminate their roles. Nursing is a humane profession and students need to be supervised, especially as there is no room for errors in practice.

## Conclusions

In summary, ICT is not a remedy that is going to solve all educational problems. However, the presence of ICT means that “there is no going back” in the educational systems available. ICT presents an essential tool for teaching and research. The current trend in higher education is that academicians are career teachers. This commitment toward education requires teachers to be committed to continuous innovation, synchronous with developments, and willing to further enrich the educational process. Despite the attitudes of nursing faculty members revealed in this study. ICT is considered important for all parties involved—faculty members, students, nurses, institutions of higher education, and healthcare providers. It has created wide access to information, and it is an essential communication tool. Although ICT is used by nursing faculty members, there is a need to update faculty members’ knowledge on the use of ICT and to provide further training. This generation of students is interested in ICT use and engaged in social media. As a result, ICT use should be incorporated into nursing curricula. Institutions can play a role in enhancing the choice of technology by developing further online courses and online degrees. Most healthcare settings are advancing and becoming more technology-oriented, and that is why it is essential for nursing faculty members to be prepared to manage different ICTs, understand their attitudes toward ICT, face the challenges and find solutions.

### Recommendations

The findings of this study have several implications for practice. First, to understand the attitudes of nursing faculty members toward the use of ICT, and second, to enhance the use of ICT in nursing teaching and learning.

### Practical implications

This study can help university administrators and faculty members to consciously increase ICT usage by addressing the challenges identified. Many barriers to the use of ICT in nursing education have been identified and revealed by faculty members. Working toward eliminating or decreasing these barriers can encourage faculty members to use ICT further in their education of nursing students [60].

Faculty members are frequently proficient in ICT use due to the infiltration of ICT into society and culture; however, their skills are developed through personal interests and need to be built upon. As a result, nursing faculty members need training on how to integrate ICT in their teaching and learning practices [37]. The development of clear ICT policies at the institutional level, the development of *e-learning* policies at the national level, and the revision of nursing curricula at the institutional level are required to create time and resources for instructors to invest in learning and managing ICT.

ICT skills are not inevitably transformed into instructional processes; hence, ICT policies should be investigated further. Institutions are encouraged to foster a culture that enhances the use of ICT because in these environments, students have better learning outcomes and instructors have better motivation levels [13].

### Future research

Demographic characteristics and individual differences should be further investigated in future studies to explore age and gender differences in *e-learning*. Pedagogic methods of teaching that use ICT also merit further study. Different faculties require capacity building, so they are at ease when using ICT. This requires resources and it is not easy [61]. Faculty members should be encouraged to attend local and international meetings, workshops, and conferences, so that they are exposed to different ICT practices in nursing education, leading to a change in attitude and increased receptivity toward using ICT.

Since the findings of this study are not generalizable to the national level, it is important to further investigate critical factors that affect attitudes toward ICT use in individual settings, so that further challenges are highlighted and recommendations can be more generalizable.

Finally, it would be interesting to compare the findings of this study, with similar studies done among participants of other disciplines or in other contexts. Therefore, different attitudes can be further analyzed, and different challenges highlighted, leading to better utilization of ICT by faculty members in different domains [60]. ICT will continue to progress in the future, and this generation of students is ICT-oriented, so educators should embrace ICT in their learning and teaching methodologies.

## Data Availability

Hard copies of the survey instruments are available with the author. The recorded interviews are available in the authors’ PC and the transcriptions of the interviews are available on A8 sheets. Data are available from the corresponding author upon request.

## Abbreviations

ICT: Information and Communication Technology
LCMS: Learning content management systems
HIS: Health information systems
MEHE: The Ministry of Education/Higher Education
MMR: Mixed Methodology Research
PCs: Personal computers
WHO: The World Health Organization
VPREC: Virtual Programme Research Ethics Committee
